# Analysis of related factors for neuropsychiatric comorbidities in children with epilepsy

**DOI:** 10.1186/s40001-024-01751-5

**Published:** 2024-03-12

**Authors:** Xin-Ying Zhang, Meng Sun, Jiang-Ya Wang, Fang-Fang Du, Xue-Fei Liu, Ling-Jun Wang, Zhen-De Hou, Ya-Ying Cheng

**Affiliations:** Department of Pediatrics, HeBei General Hospital, No.348 of Heping West Road, Xinhua District, Shijiazhuang, 050051 China

**Keywords:** Children, Cognitive impairment, Epilepsy, Psycho behavioral disorder, Risk factors

## Abstract

**Objective:**

To analyze the risk factors affecting psychiatric behavior and study the psychobehavioral conditions of children with epilepsy.

**Method:**

We randomly selected and enrolled 294 children with epilepsy who visited and were hospitalized in the pediatric clinic of Hebei General Hospital between January 2017 and January 2022, as the study participants. We comprehensively assessed their cognitive functions using the Gesell development schedule or Wechsler Intelligence Scales. The participants were divided into the study group (*n* = 123) with cognitive impairment and the control group (*n* = 171) with normal cognitive functions, for analysis.

**Results:**

There were statistically significant differences between the two groups in disease course, frequency of epilepsy, status epilepticus, and the number of antiseizure medications (ASMs) used (*P* < 0.05), while there were no statistically significant differences in age, gender, age of onset, form of onset, interictal epileptiform discharge, history of febrile convulsion, and the time from onset to initial visit (*P* > 0.05). Based on multivariate logistic regression analysis, the course of disease, frequency of onset, status epilepticus and number of ASMs used were identified as high-risk factors for cognitive impairment in children with epilepsy. Similarly, early onset, long course of disease, known etiology, and combination of multiple drugs have a negative impact on behavioral problems, school education, and social adaptability.

**Conclusion:**

The course of disease, the frequency of onset, status epilepticus, and the number of ASMs used are high-risk factors for cognitive impairment in children with epilepsy, which can be prevented and controlled early. When selecting ASMs, their advantages and disadvantages should be weighed. Moreover, the availability of alternative treatment options must be considered. With the help of genomic technology, the causes of epilepsy should be identified as early as possible, and precision medicine and gene therapy for children with epilepsy should be actively developed.

## Introduction

Epilepsy is a sudden, brief, stereotypical, and recurrent brain disorder, characterized by a persistent tendency to epileptic seizure. According to World Health Organization (WHO) statistics, there are approximately 70 million patients with epilepsy [1]; China’s epidemiological survey revealed that the overall prevalence rate of epilepsy is 7‰, and the annual incidence rate is approximately 25/100,000. Presently, there are more than 9 million patients with epilepsy in China, and approximately 60% of the patients had epileptic seizures in their childhood [2]. Childhood is a critical period of physical, mental and brain development, and although epilepsy is a common neurological condition, it can have a much greater impact on paediatric patients than an epileptic seizure [3]. In addition, it can also affect the cognitive function, psychiatric behavior, social skills, and other aspects of life. These neuropsychiatric comorbidities are common in children with epilepsy, with a prevalence of approximately 20%–60% [4, 5], and have serious negative effects on the long-term quality of life. Therefore, early diagnosis and treatment of comorbidities and identification of factors associated with a poor prognosis are critical to the long-term management of children with epilepsy. Multiple factors, including the underlying etiology, genetic background, epilepsy, and the functions of antiseizure medications (ASMs) may affect these comorbidities. Extensive studies have been conducted in this field and a number of risk factors have been identified, such as early onset, long course of disease, and the combination of multiple ASMs [6]; however, the majority of studies focus on adults and school-aged children, and there are many factors that remain unknown. The advances in genomic technologies and widespread utilization of second-generation gene sequencing technologies in clinical practice have provided a more precise basis for the study of epileptic genetics in recent years, in addition to providing new opportunities for the study of neuropsychiatric comorbidities [7]. In this study, the clinical characteristics and genetic background of children with epilepsy at the early onset stage were analyzed to determine the relevant factors affecting cognitive functions, behavioral problems, and social interaction, and raise awareness of neuropsychiatric comorbidities of epilepsy, thus providing a basis for guiding clinical control and rational intervention, and improving the quality of life of children with epilepsy.

## Data and methods

### Clinical data

We randomly selected and enrolled 294 children with epilepsy admitted to Hebei General Hospital for pediatric outpatient and inpatient treatment, between January 2017 and January 2022.

Inclusion criteria: Epilepsy onset and classification criteria stipulated by the International League Against Epilepsy (ILAE) in 2017 [8], epilepsy diagnosis standard established by the ILAE in 2014 [9], and epilepsy confirmed by clinical presentation, video EEG, and cranial MRI.

Exclusion criteria: (1) Combined with other diseases that affect cognitive functions; (2) A history of substance abuse; (3) Congenital heart disease; (4) Severe rash, gastrointestinal reactions and other pharmaceutical side effects; (5) Unknown previous medical history and incomplete clinical data; (6) Poor patient compliance. The cognitive development and functions of pediatric patients were comprehensively assessed by neurodevelopmental pediatricians using the Gesell development schedule or Wechsler Intelligence Scales. The children were divided into two groups, the study group (*n* = 123) with intellectual disability and cognitive impairment, and the control group (*n* = 171) with normal mental development and cognitive functions. The study group consisted of 74 males and 49 females, aged 1–14 years old, with an average age of 5.59 ± 3.88. The control group consisted of 101 males and 70 females aged 1–14 years old, with an average age of 5.12 ± 3.67. There was no statistical difference between the baseline data of the two groups (*P* > 0.05), and they were comparable. This study was approved by the Ethics Committee of Hebei General Hospital, and the informed consent was obtained from the parents of the children.

### Methods

Pediatric neurologists collected clinical data, which included demographic characteristics (age, gender, educational background), clinical characteristics of the patients (cause of epilepsy, age of onset, course of disease, frequency of onset, form of onset, interictal epileptiform discharge, family history of epilepsy, previous history (history of febrile convulsion), number of ASMs used, the time from onset to first visit), findings from EEG and neuroimaging, and information on behavioral problems and social interaction. The psychiatric behavior of the pediatric patients was assessed by neurodevelopmental pediatricians using the Child Behavior Checklist (CBCL). Behavioral problems include aggressiveness, defiance, hyperactivity, and inattention. Neurodevelopmental pediatricians assessed the social adaptability of the pediatric patients using the S-M scale, and the parents were asked to clarify various living abilities and social interactions.

### Statistical analysis

SPSS 25.0 software was utilized for data processing. The normally distributed measurement data are expressed by mean ± standard deviation (*x* ± *s*), the comparison between the groups was conducted by t-test based on independent samples. The non-normally distributed measurement data are expressed as the median (interquartile range) [P50 (P25, P75)], and the Wilcoxon rank sum test was used to compare the groups. The counting data are expressed as a percentage (%), and the rates were compared by chi-squared test or the Fisher’ exact test. The correlations between various influential factors and cognitive impairment were analyzed using Pearson’s correlation analysis and Spearman’s correlation analysis. The statistically significant factors were included in the binary logistic regression analysis. *α* = 0.05 indicated the significance level, and *P* < 0.05 indicated that the difference was statistically significant.

## Results

### Basic information of children with epilepsy

**Fig. 1:**
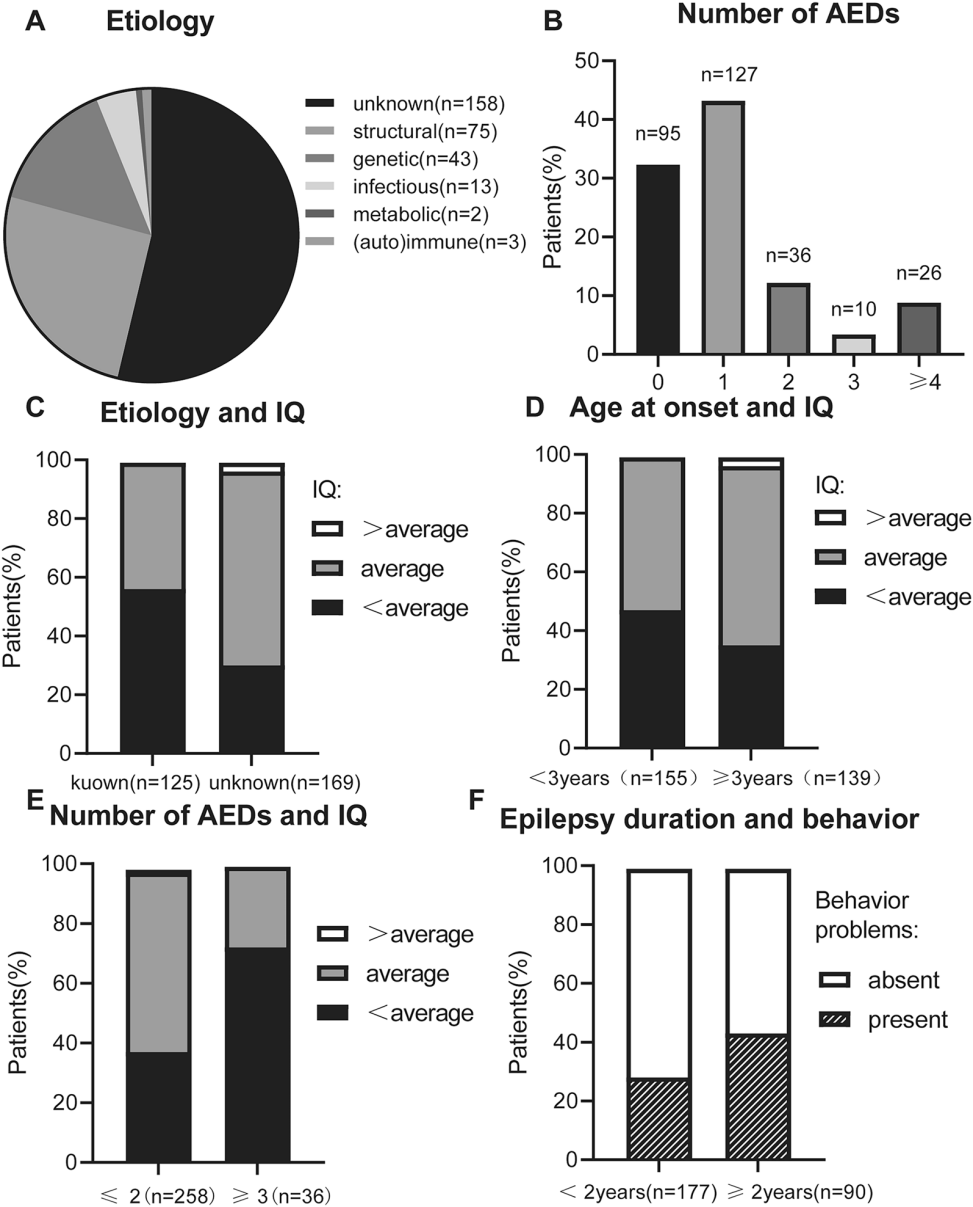
Epilepsy-related variables and correlation with cognition and behavior. **A** Distribution of known causes in children with epilepsy. **B** The proportion of antiseizure medications (ASMs) in children with epilepsy. **C** Known etiology of epilepsy is negatively correlated with cognitive function. **D** Early onset age is negatively correlated with cognitive function. **E** The number of ASMs is negatively correlated with cognitive function. **F** Behavioral problems increase with the increase in duration of epilepsy

The most common type of epilepsy in children is generalized seizure with a rate of 79.6% and an interictal discharge rate of 58.8%, mainly during sleep. Among the 294 children with epilepsy, 59.5% were male while 40.5% were female. The mean age of the pediatric patients was 5.32 ± 3.76 years, and the average duration of their epilepsy was 1.51 ± 2.30 years. Figure 1A shows the distribution of commonly known causes of epilepsy in children. Figure 1B shows the number of ASMs given to children with epilepsy. Table 1 shows statistics on specific variables of epilepsy in children. Tables 2 and 3 show the distribution of the most commonly known causes of epilepsy in children.

**Table 1:** Statistics on specific variables of epilepsy in children

Variables	Grouping	Study group, *n* (%)	Control group, *n* (%)	*t*/*z*/*χ*^2^	*P*-value
Age (years)		6.00 (6.42)	5.00 (6.17)	− 1.015	0.310
Gender *n* (%)	Female	49 (39.8%)	70 (40.9%)	0.036	0.850
	Male	74 (60.2%)	101 (59.1%)		
Age of onset		2.00 (5.31)	2.83 (5.00)	− 1.272	0.203
Course of disease		0.92 (2.98)	0.25 (1.99)	− 2.316	0.021
Frequency	≥ once/day	46 (37.4%)	33 (19.3%)	26.693	0.000
	≥ once/week	23 (18.7%)	18 (10.5%)		
	≥ once/month	18 (14.6%)	20 (11.7%)		
	≥ once/year	33 (26.8%)	95 (55.6%)		
	< once/year	3 (2.4%)	5 (2.9%)		
Form of onset	Generalized onset	100 (81.3%)	140 (81.9%)	0.016	0.901
	Focal onset	23 (18.7%)	31 (18.1%)		
Status epilepticus	Yes	38 (30.9%)	29 (17.0%)	7.895	0.005
	No	85 (69.1%)	142 (83.0%)		
Interictal epileptiform discharge	Yes	74 (60.2%)	99 (57.9%)	0.152	0.697
	No	49 (39.8%)	72 (42.1%)		
Family history	Yes	27 (22.0%)	33 (19.3%)	0.310	0.578
	No	96 (78.0%)	138 (80.7%)		
History of febrile convulsions	Yes	28 (22.8%)	36 (21.1%)	0.123	0.726
	No	95 (77.2%)	135 (78.9%)		
Birth history and brain injury abnormalities	Yes	48 (38.2%)	24 (14.0%)	22.828	0.000
	No	75 (61.8%)	147 (86.0%)		
Number of antiepileptic drugs	No medication	30 (24.4%)	65 (38.0%)	19.364	0.001
	Single	50 (40.7%)	77 (45.0%)		
	Two	17 (13.8%)	19 (11.1%)		
	Three	6 (4.9%)	4 (2.3%)		
	Three and above	20 (16.3%)	6 (3.5%)		
Time from onset to first visit		0.02 (0.50)	0.02 (0.67)	− 0.668	0.504

**Table 2:** Common structural causes of pediatric epilepsy

Structural causes	*n*
White matter dysplasia	9
Encephalatrophy	4
White matter myelination insufficiency	15
Agenesis of corpus callosum	9
Cortical dysplasia	11
Leukomalacia	9
Demyelinating disease	6
Hydrocephalus	3
Delayed myelination (white matter)	6
Sequelae of viral encephalitis	1
Hypoxic-ischemic brain injury	1
Subdural effusion	1
Subdural hemorrhage	1
Widening and dilatation of the ventricle and lateral ventricle	11
Basilar invagination	1
Tuberous sclerosis	3
Hippocampal sclerosis (focal cortical dysplasia FCD Ib)	1
Cerebral dysplasia	4
Hippocampal dysplasia	5
Macrogyria	2
Polygyri	1
Gray matter shift	1

**Table 3:** Common genetic causes in children with epilepsy

Genetic causes	*n*
Tuberous sclerosis (TSC gene mutation)	3
Angel syndrome/pradar-Willi syndrome (UBE3A gene heterozygous deletion)	2
Dravet syndrome (SCN1A gene mutation)	2
Infantile epilepstic spasms syndrome (IESS) (trisomy 21, ARX gene mutation, CDKL5 gene mutation, STXBP1 gene mutation, IQSEC2 gene mutation, TSC1 gene mutation, and TSC2 gene mutation)	7
Trisomy 21 syndrome (karyotype analysis: 47, XY + 21)	1
Methylmalonic acidemia with hyper homocysteinemia	2
Y chromosome long arm deletion (deletion greater than 3372 Kb of Y chromosome long arm)	1
Karyotype analysis: 46, XY (Y is equivalent to 18)	1
SCN1A gene mutation (unclear clinical phenotype)	1
SCN2A gene mutation	1
SCN8A gene mutation	1
KCNO2 gene mutation	3
FASN gene mutation	1
PRRT2 gene mutation	1
KCNT1 gene mutation	1
PARS2 gene mutation	1
GRIN2B gene mutation	1
RHOBTB2 gene mutation	1
CDKL5 gene mutation	1
PCDH19 gene mutation	1
GABRB3 gene mutation	1
GABRA1 gene mutation	1
STXBP1 gene mutation	1
SLC35A2 gene mutation	1
UBA5 gene mutation	1
PACS1 gene mutation	1
ARID1B gene mutation	1
BRANBP2 gene mutation	1
ANKRD11, NOTCH1 gene mutation	1

### Influencing factors of cognitive impairment

#### Univariate analysis of intellectual disability and cognitive impairment among children with epilepsy

Table 1 displays a comparison of the basic data and epilepsy onset-related data between the two groups; there were no statistically significant differences in age, gender, age of onset, form of onset, interictal epileptiform discharge, history of febrile convulsion, and the time from onset to first visit between the two groups (*P* > 0.05), however, there were statistically significant differences in the disease course, onset frequency, status epilepticus, and number of ASMs used (*P* < 0.05).

#### Multivariate logistic regression analysis of intellectual disability and cognitive impairment in children with epilepsy

Table 4 displays a comparison of the data related to the onset of epilepsy between the two groups; there were statistically significant differences in progression of the disease, frequency of onset, status epilepticus, and number of ASMs used (*P* < 0.05).

**Table 4:** Multivariate logistic regression analysis of cognitive impairment in children with epilepsy

Variables	B	Standard error	Wald	Degree of freedom	Significance	Exp (B)	95% CI
Course of disease	− 0.216	0.061	12.470	1	0.000	0.806	0.714–0.908
Frequency	− 0.549	0.171	10.245	1	0.001	0.578	0.413–0.808
Status epilepticus	1.408	0.557	6.389	1	0.011	4.089	1.372–12.186
Number of antiepileptic drugs	− 0.452	0.228	3.929	1	0.047	0.636	0.407–0.995
Constant	2.086	0.345	36.515	1	0.000	8.050	

Mental development and cognitive function were negatively associated with epilepsy of known etiology, early onset epilepsy, and the combined usage of multiple ASMs. In this study, the cause of epilepsy was found to be strongly associated with mental development and cognitive function: compared to children with epilepsy of unknown etiology, children with epilepsy of known etiology were more likely to have intellectual disability and cognitive impairment (*χ*^2^ = 21.826, *P* = 0.000; Fig. 1C).

Similarly, the earlier onset age had a greater impact on mental development and cognitive function of children. Mental development and cognitive function were more likely to be below the average in children with a history of epilepsy prior to the age of three, compared to those who had epilepsy after the age of three (*χ*^2^ = 9.184, *P* = 0.006; Fig. 1D).

Mental development and cognitive function were negatively correlated with the number of ASMs used. Compared to pediatric patients taking two or fewer ASMs, those taking three or more ASMs are more likely to have intellectual disability and cognitive impairment (*χ*^2^ = 14.749, *P* = 0.000; Fig. 1E).

### Factors affecting behavioral problems

The behavioral data of the 267 pediatric patients were collected. Behavioral problems were present in 33.7% (*n* = 90) of pediatric patients. The most significant symptoms were aggression and self-aggression, accounting for 47.2% (*n* = 42), followed by attention deficit, accounting for 33.7% (*n* = 30). It was found that the duration of epilepsy had a negative impact on behavior: The incidence of behavioral problems increased with the increase in duration (*χ*^2^ = 5.629, *P* = 0.018; Fig. 1F).

### Factors affecting school education and social interaction

The educational data of 192 pediatric patients were collected. Among the study participants, 37.5% (*n* = 72) went to regular schools, and 62.5% (*n* = 120) went to special educational institutions. In this study, the compounded medication (> 3 ASMs) (*χ*^2^ = 20.496, *P* = 0.000), duration of epilepsy (> 3 years) (*χ*^2^ = 10.678, *P* = 0.001), early age of onset (< 3 years) (*χ*^2^ = 10.844, *P* = 0.001) and epilepsy of known etiology (*χ*^2^ = 17.844, *P* = 0.000) were identified as the risk factors for special educational needs. The data on social adaptability of the 277 pediatric patients were collected. In 44.4% (*n* = 123) of pediatric patients, the social adaptability was consistent with age, while in 55.6% (*n* = 154) of pediatric patients, the social adaptability was impaired. Impaired social adaptability was associated with early onset (< 3 years) (*χ*^2^ = 10.313, *P* = 0.001), known etiology (*χ*^2^ = 17.500, *P* = 0.000), and compounded medication (> 3 ASMs) (*χ*^2^ = 33.084, *P* = 0.000).

## Discussion

Epilepsy is a common chronic disease of the nervous system. The brains of children are at an early stage of growth and development, without mature brain functions. Thus, repeated epileptic seizures can lead to impaired or even regressed brain development [10]. According to a study, 30%–40% of patients with epilepsy had intellectual disability, and more cognitive impairments and psychobehavioral comorbidities than normal children, which could lead to learning impairment and social dysfunction, thus seriously affecting the quality of life of the children with epilepsy [11]. Cognitive impairment and psycho behavioral comorbidities are often overlooked in the diagnosis and treatment of epilepsy, and even children with controlled seizure have poor long-term social prognosis [12]. Therefore, it is important for clinical prevention and treatment to focus on cognitive impairment and psychobehavioral comorbidities in children with epilepsy, and actively explore the early predictors of cognitive impairment and psychobehavioral comorbidities.

The results of this study demonstrated the negative impact of epilepsy on cognition, behavior, education, and social adaptability. It was found that ASMs, the early age of onset, epilepsy types (especially those of known etiology), duration of epilepsy, and combination of multiple ASMs negatively impacted cognition, behavior, education, and social adaptability, which is consistent with the results of previous studies [13, 14]. Previously, clinicians attributed cognitive impairment in pediatric patients to the side effects of ASMs, recurrent epilepsy, different epilepsy types, and the influence of psychosocial factors due to long-term illness. Studies have shown that the frequency of onset, form of onset, types of ASMs used, course of disease and EEG status were independent risk factors of cognitive impairment [15, 16]. In Finland, a 50-year prospective cohort study showed that long-term persistent epilepsy and anti-epileptic medication had a significant role in long-term cognitive outcomes of children with epilepsy, significantly increasing the risks of intellectual disability and cognitive impairment. A large cohort study of 371 children at the University of Berlin Medical School in Germany found that early onset epilepsy, epilepsy of known etiology, and multidrug therapy were most likely to present with cognitive impairment [17]. Most neuropsychiatric and psychosocial factors, such as age of onset or underlying etiology, are immutable. Therefore, it is only possible to control the use of multiple ASMs. Although ASMs can alleviate epilepsy, and have a positive effect on neuropsychiatric comorbidities, there are obvious cognitive and behavioral side effects. Therefore, ASMs should be used rationally according to the types and characteristics of epilepsy, their advantages and disadvantages should be weighed, and individualized monotherapy, rather than drug combinations should be utilized based on the principle of controlling epilepsy with the smallest possible dose; combining more than two ASMs should be avoided, and the drugs should be stopped as early as possible when the safety of pediatric patients can be ensured. Witt et al. found that cognition and behavior can be improved by keeping the drug load as low as clinically permissible, and reducing or stopping the use of ASMs as early as possible; in terms of drug selection, drugs with fewer cognitive and behavioral side effects should be selected, such as lamotrigine, levetiracetam, and lacosamide, rather than topiramate, phenytoin, and phenobarbital that have greater side effects [18]. Boshuisen et al. showed that fewer ASMs taken after pediatric epilepsy surgery can improve the level of intelligence in children with epilepsy [19]. Lamberink et al. showed that heavy ASMs usage was an independent risk factor for poor long-term prognosis in patients with epilepsy [20]. Early identification of genetic causes, such as ion channelopathies, can guide the selection of ASMs and reduce the combined treatment with multiple ASMs. For children with symptomatic epilepsy, the long duration of epilepsy and the combination of multiple ASMs result in poor prognosis; therefore, the possibility of epilepsy surgery should be assessed at an early stage of disease. Despite the fact that the efficacy of epilepsy surgery has been demonstrated in multiple studies, there would typically be a long course of disease prior to the epilepsy surgery (16 years on average, and 5.3 years for children) [21, 22]. Despite the clear correlation, it has not been applied to the daily prevention and care of clinical patients. Future studies should confirm the potential associations between the factors observed in this retrospective study.

## Conclusion

The combination of multiple ASMs is the only modifiable risk factor for resolving the poor cognitive and psychobehavioral outcomes. During the treatment of epilepsy, the advantages and disadvantages of the combination of ASMs should be weighed; the combination of ASMs should be minimized when permitted, and drug replacement therapies, such as epilepsy surgery, vagus nerve stimulation, hormone therapy and ketogenic diet therapy should be considered as early as possible. With the advances in genomic technologies and widespread use of second-generation gene sequencing technology in recent years, we have improved our understanding of epilepsy, found that genetic factors are important factors affecting the occurrence of epilepsy, and improved the possibility of developing precision medicine and gene therapy for children with epilepsy, so for children with epilepsy with clear etiology, the cause treatment should be started as soon as possible. Children with intractable epilepsy should also be referred to an epilepsy center or experienced specialist hospital for treatment.

## Limitations

We acknowledge this study has limitations. Namely, the cognitive and developmental profile of children and adolescents with epilepsy depends on a complex interaction between multiple factors. This could somehow influence our results. For example, the correlation between ASMs and cognitive profile in children with epilepsy and which ASM have a higher correlation, the impact of environmental background (educational level of the parents, socio-environmental and socio-cultural factors within and outside the family) on cognitive and developmental profile of children and adolescents with epilepsy, and the influence of genetic polymorphisms on drug resistant epilepsy in pediatric patients and on cognitive and developmental profile of children and adolescents with epilepsy. These factors should be evaluated in follow-up studies. However, the large sample size and longitudinal nature of the study, along with a comprehensive neuropsychological test battery, with several cognitive domains assess.

## Data Availability

The datasets used and/or analyzed during the current study available from the corresponding author on reasonable request.

## Abbreviations

WHO: World Health Organization
ASMs: Antiseizure medications
ILAE: International League against Epilepsy
CBCL: Child Behavior Checklist
EEG: Electroencephalo-graph
MRI: Magnetic resonance imaging
